# Subsequent Risk of Metabolic Syndrome in Women With a History of Preeclampsia: Data From the Health Examinees Study

**DOI:** 10.2188/jea.JE20140136

**Published:** 2015-04-05

**Authors:** Jae Jeong Yang, Sang-Ah Lee, Ji-Yeob Choi, Minkyo Song, Sohee Han, Hyung-Suk Yoon, Yunhee Lee, Juhwan Oh, Jong-Koo Lee, Daehee Kang

**Affiliations:** 1 Department of Preventive Medicine, Seoul National University College of Medicine, Seoul, Korea; 2 Institute of Environmental Medicine, Seoul National University Medical Research Center, Seoul, Korea; 3 Department of Preventive Medicine, Kangwon National University School of Medicine, Kangwon, Korea; 4 Department of Biomedical Sciences, Seoul National University Graduate School, Seoul, Korea; 5 Cancer Research Institute, Seoul National University, Seoul, Korea; 6 JW LEE Center for Global Medicine, Seoul National University College of Medicine, Seoul, Korea; 7 Department of Family Medicine, Seoul National University College of Medicine, Seoul, Korea

**Keywords:** history of preeclampsia, metabolic syndrome, cohort, HEXA, Korea

## Abstract

**Background:**

To investigate whether preeclampsia is independently associated with risk of future metabolic syndrome and whether any such primary associations are modified by different ages at first pregnancy.

**Methods:**

Based on the Health Examinees Study, a cross-sectional analysis was conducted. Data of women (*n* = 49 780) who had experienced at least 1 pregnancy during their lifetime and had never been diagnosed with any metabolic disorder before their pregnancy were analyzed using multiple logistic regression models. Odds ratios (ORs) and 95% confidence intervals (CIs) were estimated after adjusting for age, lifestyle characteristics, and reproductive factors. A stratified analysis was also conducted to estimate the extent of the primary association between preeclampsia and future metabolic syndrome by age at first pregnancy.

**Results:**

Women with a history of preeclampsia had significantly increased odds of developing metabolic syndrome (adjusted OR 1.23; 95% CI, 1.12–1.35), central obesity (adjusted OR 1.36; 95% CI, 1.25–1.47), elevated blood pressure (adjusted OR 1.53; 95% CI, 1.41–1.67), or elevated fasting glucose (adjusted OR 1.13; 95% CI, 1.03–1.25) in later life. In the stratified analysis, women who first became pregnant at ages >35 years and had preeclampsia were found to be at significantly increased likelihood of metabolic syndrome later in life (adjusted OR 4.38; 95% CI, 1.62–11.9).

**Conclusions:**

Our findings suggest that preeclampsia increases the risk of metabolic syndrome in later life, and late age at first pregnancy can further exacerbate this risk.

## INTRODUCTION

Preeclampsia is one of the most common complications of pregnancy. This pregnancy-specific syndrome, characterized by new-onset hypertension along with proteinuria during gestation, occurs in approximately 3%–8% of all pregnancies worldwide.^1^ Preeclampsia is generally regarded as a disease of the first pregnancy, but it frequently recurs in later pregnancies.^2^^–^^4^ Preeclampsia increases the risk of maternal mortality, as well as neonatal morbidity and/or mortality.^1^^,^^5^ Furthermore, compelling evidence indicates that the long-term effects of preeclampsia are associated with an increased risk of cardiovascular disease in later life.^3^^,^^6^^–^^10^

The underlying mechanism for the transition from preeclampsia to cardiovascular disease appears to be multifactorial but has not yet been fully elucidated. Previous studies have suggested that preeclampsia is associated with pathophysiological abnormalities, including endothelial dysfunction and systemic hypertension, and metabolic disorders, such as obesity, insulin resistance, hyperglycemia, and diabetes mellitus.^3^^,^^6^^,^^11^^–^^14^ The occurrence of a cluster of these perturbations in multiple metabolic pathways is generally known as metabolic syndrome, a condition that is assumed to create a milieu leading to increased risk of cardiovascular disease.^15^ This means that preeclampsia can increase the risk of metabolic syndrome, which may be an ascendant event of future cardiovascular disease.

In the disease trajectory from preeclampsia to cardiovascular disease, metabolic syndrome may play a crucial role as a putative mediator and/or indicator of susceptibility. It is possible that preeclamptic women can easily progress to metabolic syndrome, which is generally defined as a cluster of cardiovascular risk factors characterized by insulin resistance,^16^^,^^17^ or that a subclinical susceptibility to future metabolic syndrome may predispose women to preeclampsia.^18^ We therefore hypothesized that preeclampsia may be a risk factor for metabolic alterations that lead to the development of cardiovascular disease later in life. Furthermore, given that female reproductive history is considered a component of the etiology of several chronic diseases, including metabolic syndrome,^19^ the association between preeclampsia and future metabolic syndrome could be mediated by certain pregnancy-related factors, particularly age at first pregnancy. To explore this hypothesis, a cross-sectional study, based on data from a large-scale cohort study, was conducted in a Korean population to investigate: 1) whether preeclampsia is independently associated with increased risk of metabolic syndrome and its components later in life, and 2) whether any such primary associations are modified by age at first pregnancy.

## MATERIALS AND METHODS

### Study population

The present study is based on a large-scale genomic cohort study, the Health Examinees (HEXA) Study, which was conducted in Korea. Middle-aged and elderly people were prospectively recruited for the HEXA study following a standardized study protocol, which was approved by the Ethics Committee of the Korean Health and Genomic Study of the Korean National Institute of Health and the institutional review boards of all participating hospitals. Before entering the study, all participants voluntarily signed an informed consent form. An interview-based questionnaire survey was conducted to collect information on individual characteristics, including socio-demographic factors, disease history, lifestyle, and dietary habits. Biological samples (ie, plasma, serum, buffy coat, blood cells, genomic DNA, and urine) were collected at baseline and stored under stable conditions. Physical examinations and laboratory analyses were performed by trained medical staff.

The study samples were selected as follows: from a total of 85 323 subjects aged 40–69 years who participated in the HEXA study between 2004 and 2008, men (*n* = 28 075) and nulligravida women (*n* = 1902) were excluded. Eligible women who had experienced at least 1 pregnancy during their lifetime were preliminarily screened (*n* = 55 346). Of these, we excluded women for whom the following information was not available: preeclampsia history (*n* = 1026), age at first pregnancy (*n* = 259), past/current history of metabolic disorders (ie, hypertension, hyperlipidemia, or diabetes mellitus; *n* = 105), and any kind of anthropometry measures (ie, waist circumference, systolic/diastolic blood pressure, and levels of triglyceride, fasting blood glucose, and high-density lipoprotein [HDL] cholesterol; *n* = 3307). To avoid the problem of temporal ambiguity, we also excluded women who had been diagnosed with any metabolic disorder (ie, hypertension, hyperlipidemia, or diabetes mellitus) before their first pregnancy (*n* = 869). After exclusions, a total of 49 780 women were included in the final analysis.

### Definition of history of preeclampsia

History of preeclampsia was evaluated by posing questions in phases. First, women were classified as either gravida or nulligravida according to their response to the question “Have you ever been pregnant?” Nulligravida women were excluded from the analysis. The gravida women were then asked, “At what age did you first become pregnant?” and “Have you ever been diagnosed with preeclampsia by a doctor in a hospital?” Women who responded “yes” to the last question were assigned to the preeclampsia-positive group; the respondents who answered “no” were assigned to the preeclampsia-negative group. If a woman was unsure of her history of preeclampsia, she was excluded from the analysis.

### Definition of metabolic syndrome

Metabolic syndrome and its individual components were defined according to the National Cholesterol Education Program Adult Treatment Panel III (NCEP-ATP III) criteria. The NCEP-ATP III criteria for metabolic syndrome require the presence of 3 or more of the following^20^: a waist circumference ≥80 cm, systolic blood pressure ≥130 mm Hg and/or diastolic blood pressure ≥85 mm Hg or consuming antihypertensive medication, fasting triglyceride levels ≥150 mg/dL or consuming antihyperlipidemic medication, fasting plasma glucose ≥100 mg/dL or consuming antidiabetic medication, and fasting HDL cholesterol <50 mg/dL or consuming anticholesterol medication.

### Statistical analysis

The chi-squared test and Student’s *t*-test were used to compare the basic characteristics of gravida women who had experienced a pregnancy complicated by preeclampsia and those who had not. All results with a *P*-value <0.05 were considered statistically significant.

To investigate whether preeclampsia was independently associated with risk of subsequent metabolic syndrome and its individual components, 3 hierarchical multivariate logistic regression models were used to estimate odds ratios (ORs) and 95% confidence intervals (CIs) accounting for: age at enrollment (Model 1); age at enrollment, age at first pregnancy, number of childbirths, BMI before pregnancy, history of spontaneous abortion, history of artificial abortion, breastfeeding experience, use of oral contraceptives, and menopausal status (Model 2); and age at enrollment, age at first pregnancy, number of childbirths, BMI before pregnancy, history of spontaneous abortion, history of artificial abortion, breastfeeding experience, use of oral contraceptives, menopausal status, education level, smoking status, drinking status, and physical exercise (Model 3). Furthermore, to estimate the primary association between preeclampsia and metabolic syndrome by age at first pregnancy, a stratified analysis (age >35 years, age 31–35 years, and age ≤30 years) was conducted in the same manner. In the stratified analysis, to determine whether the subsequent risk of metabolic syndrome increased with age at first pregnancy, trend tests were performed. Interaction effects between preeclampsia and age at first pregnancy on metabolic syndrome and its components were also evaluated using multiplicative interaction terms in the multivariate logistic regression models. All other statistical analyses were performed using SAS software version 9.3 (SAS Institute, Cary, NC, USA).

## RESULTS

Characteristics of study participants regarding lifestyle, reproduction, and anthropometry are summarized in Table 1. When all putative confounders were accounted for, women with metabolic syndrome were more likely to be older, less educated, nondrinkers, and non-regular exercisers, and to have a history of preeclampsia, be younger at their first pregnancy, and have had less experience with artificial abortion (*P* < 0.001) (Table 1). Women diagnosed with preeclampsia tended to be older at first pregnancy (25.4 years vs 24.9 years) and have higher rates of spontaneous (27.9% vs 23.3%) and artificial (71.2% vs 66.3%) abortions; these differences remained statistically significant after adjusting for potential confounders. When all putative confounders were taken into consideration, women diagnosed with preeclampsia were found to have a significantly higher mean waist circumference (*P* < 0.05) (Table 2).

**Table 1.** Basic characteristics of the study population across metabolic syndrome (*n* = 49 780)

	Metabolic syndrome (*n* = 12 687)	Non-metabolic syndrome (*n* = 37 093)	*P*^a^	*P*^b^	*P*^c^
Life style factors					
Age, years	56.5 (7.2)	51.1 (7.3)	<0.001	<0.001	<0.001
≥High school graduation	4734 (37.3)	22 490 (60.6)	<0.001	<0.001	<0.001
Never smokers	12 215 (96.3)	35 626 (96.1)	0.242	0.149	0.251
Never drinkers	9399 (74.1)	24 184 (65.2)	<0.001	<0.001	<0.001
Regular exercisers	5962 (47.0)	19 158 (51.7)	<0.001	<0.001	<0.001
Reproductive factors					
History of preeclampsia	708 (5.6)	1882 (5.0)	<0.001	<0.001	<0.001
Age at first pregnancy, years	24.3 (3.2)	25.2 (3.2)	<0.001	<0.001	<0.001
Number of childbirths	2.8 (1.2)	2.3 (0.9)	0.853	0.320	0.439
History of spontaneous abortion	2963 (23.4)	8771 (23.7)	0.119	0.222	0.280
History of artificial abortion	8269 (65.2)	24 842 (67.0)	0.002	<0.001	<0.001
Breastfeeding experience	11 517 (90.8)	31 790 (85.7)	0.040	0.517	0.767
Use of oral contraceptives	3160 (24.9)	7213 (19.5)	0.006	<0.001	0.003
Menopause	9588 (75.6)	18 780 (50.6)	0.179	<0.001	<0.001
Anthropometry					
Waist circumference, cm	85.5 (6.9)	77.1 (7.3)	<0.001	<0.001	<0.001
Systolic blood pressure, mm Hg	131.2 (15.9)	117.6 (14.6)	0.005	0.007	0.008
Diastolic blood pressure, mm Hg	80.8 (9.6)	73.6 (9.5)	0.005	0.007	0.009
Triglycerides, mg/dL	174.4 (97.5)	93.5 (49.1)	<0.001	<0.001	<0.001
Fasting glucose, mg/dL	104.2 (29.1)	89.3 (15.1)	<0.001	<0.001	<0.001
HDL cholesterol, mg/dL	47.7 (10.2)	58.8 (12.1)	<0.001	<0.001	<0.001

HDL, high-density lipoprotein.

Data are the mean (standard deviation) or *n* (%).

^a^Adjusted *P* values by age at enrollment.

^b^Adjusted *P* values by age at enrollment, age at first pregnancy, number of childbirths, BMI status before pregnancy, history of spontaneous abortion, history of artificial abortion, breastfeeding experience, use of oral contraceptives, menopausal status, and history of preeclampsia.

^c^Adjusted *P* values by age at enrollment, age at first pregnancy, number of childbirths, BMI status before pregnancy, history of spontaneous abortion, history of artificial abortion, breastfeeding experience, use of oral contraceptives, menopausal status, education level, smoking status, drinking status, physical exercise, and history of preeclampsia.

**Table 2.** Comparison of characteristics according to history of preeclampsia

	History of preeclampsia (*n* = 2590)	No history of preeclampsia (*n* = 47 190)	*P*^a^	*P*^b^	*P*^c^
Life style factors					
Age, years	52.0 (7.4)	52.5 (7.6)	0.003	0.012	0.017
≥High school graduation	1510 (58.3)	25 714 (54.5)	0.354	0.789	0.917
Never smokers	2477 (95.6)	45 364 (96.1)	0.041	0.275	0.811
Never drinkers	1735 (67.0)	31 848 (67.5)	0.065	0.067	0.235
Regular exercisers	1386 (53.5)	23 734 (50.3)	0.011	0.022	0.024
Reproductive factors					
Age at first pregnancy, years	25.4 (3.2)	24.9 (3.3)	<0.001	<0.001	<0.001
Number of childbirths	2.4 (0.9)	2.4 (1.0)	0.654	0.614	0.653
History of spontaneous abortion	723 (27.9)	11 011 (23.3)	<0.001	<0.001	<0.001
History of artificial abortion	1844 (71.2)	31 267 (66.3)	<0.001	<0.001	<0.001
Breastfeeding experience	2207 (85.2)	41 100 (87.1)	0.026	0.322	0.306
Use of oral contraceptives	589 (22.7)	9784 (20.7)	0.445	0.057	0.037
Menopause	1472 (56.8)	26 896 (57.0)	0.204	0.028	0.031
Anthropometry					
Waist circumference, cm	80.3 (8.3)	79.2 (8.1)	<0.001	<0.001	<0.001
Systolic blood pressure, mm Hg	123.3 (16.5)	121.0 (16.0)	0.119	0.159	0.162
Diastolic blood pressure, mm Hg	76.8 (10.4)	75.4 (10.0)	0.211	0.263	0.268
Triglycerides, mg/dL	114.7 (73.8)	114.1 (73.9)	0.292	0.188	0.150
Fasting glucose, mg/dL	93.9 (21.4)	93.1 (20.6)	0.976	0.999	0.996
HDL cholesterol, mg/dL	55.7 (12.6)	56.0 (12.6)	0.172	0.086	0.054

HDL, high-density lipoprotein.

Data are the mean (standard deviation) or *n* (%).

^a^Adjusted *P* values by age at enrollment.

^b^Adjusted *P* values by age at enrollment, age at first pregnancy, number of childbirths, BMI status before pregnancy, history of spontaneous abortion, history of artificial abortion, breastfeeding experience, use of oral contraceptives, and menopausal status.

^c^Adjusted *P* values by age at enrollment, age at first pregnancy, number of childbirths, BMI status before pregnancy, history of spontaneous abortion, history of artificial abortion, breastfeeding experience, use of oral contraceptives, menopausal status, education level, smoking status, drinking status, and physical exercise.

The prevalence of metabolic syndrome and its components among preeclamptic women is shown in Table 3. A total of 27.3% of preeclamptic women met the criteria for metabolic syndrome. More than one-third of the subjects had central obesity (51.1%), elevated blood pressure (45.1%), and reduced levels of HDL cholesterol (35.2%). Accounting for selected covariates, including lifestyle characteristics and reproductive history, women diagnosed with preeclampsia had significantly increased odds of developing metabolic syndrome (OR 1.23; 95% CI, 1.12–1.35), central obesity (OR 1.36; 95% CI, 1.25–1.47), elevated blood pressure (OR 1.53; 95% CI, 1.41–1.67), and elevated fasting glucose levels (OR 1.13; 95% CI, 1.03–1.25) in later life (Table 3).

**Table 3.** Risk of the development of metabolic syndrome and its components in women with and without a history of preeclampsia

	History of preeclampsia (*n* = 2590)	No history of preeclampsia (*n* = 47 190)	Model 1^a^ OR (95% CI)	Model 2^b^ OR (95% CI)	Model 3^c^ OR (95% CI)
Metabolic syndrome	708 (27.3)	11 979 (25.4)	1.18 (1.07–1.29)	1.22 (1.11–1.34)	1.23 (1.12–1.35)
Individual component of metabolic syndrome					
Elevated waist circumference	1323 (51.1)	21 609 (45.8)	1.30 (1.20–1.42)	1.34 (1.24–1.46)	1.36 (1.25–1.47)
Elevated blood pressure	1168 (45.1)	17 630 (37.4)	1.50 (1.38–1.63)	1.53 (1.40–1.66)	1.53 (1.41–1.67)
Elevated triglyceride	600 (23.2)	10 614 (22.5)	1.08 (0.98–1.18)	1.09 (0.99–1.20)	1.09 (0.99–1.20)
Elevated fasting glucose	583 (22.5)	10 051 (21.3)	1.11 (1.01–1.22)	1.13 (1.02–1.24)	1.13 (1.03–1.25)
Reduced HDL cholesterol	911 (35.2)	16 341 (34.6)	1.05 (0.96–1.14)	1.05 (0.97–1.15)	1.06 (0.97–1.15)

CI, confidence interval; HDL, high-density lipoprotein; OR, odds ratio.

^a^Adjusted *P* values by age at enrollment.

^b^Adjusted *P* values by age at enrollment, age at first pregnancy, number of childbirths, BMI status before pregnancy, history of spontaneous abortion, history of artificial abortion, breastfeeding experience, use of oral contraceptives, and menopausal status.

^c^Adjusted *P* values by age at enrollment, age at first pregnancy, number of childbirths, BMI status before pregnancy, history of spontaneous abortion, history of artificial abortion, breastfeeding experience, use of oral contraceptives, menopausal status, education level, smoking status, drinking status, and physical exercise.

A stratified analysis was also conducted to account for the potent effect of late age at first pregnancy (Table 4). Almost half of the women with a history of preeclampsia and late age at first pregnancy (>35 years) had metabolic syndrome (43.5%); these women had a 4.38-times higher likelihood of metabolic syndrome (95% CI, 1.62–11.9), as evaluated using the fully adjusted models. Furthermore, the respective odds of central obesity and reduced HDL cholesterol level later in life were 3.81 times (95% CI, 1.44–10.0) and 5.50 times (95% CI, 2.12–14.3) higher among these women than in those with preeclampsia who became pregnant at younger ages. Even when all putative covariates were accounted for, preeclamptic women who first became pregnant between the age of 31 and 35 years were twice as likely to develop metabolic syndrome (OR 2.12; 95% CI, 1.29–3.49), central obesity (OR 2.26; 95% CI, 1.47–3.46), and elevated blood pressure (OR 2.41; 95% CI, 1.55–3.74). Compared to women who first became pregnant at younger ages, preeclamptic women who first became pregnant at age >35 years tended to have higher odds of developing metabolic syndrome in later life. The subsequent risk of metabolic syndrome, central obesity, and elevated blood pressure increased with age at first pregnancy (*P* for trend <0.001). Significant interaction effects were observed between preeclampsia and age at first pregnancy in terms of the development of metabolic syndrome, central obesity, and reduced HDL cholesterol (*P* = 0.007, *P* = 0.006, and *P* = 0.002, respectively) (Table 4).

**Table 4.** Risk estimates for metabolic syndrome and each component of the metabolic syndrome in women with a history of preeclampsia: stratified analysis by age group at first pregnancy

	Age group at first pregnancy			*P* for trend	*P* interaction

	≤30 years old OR (95% CI)	31–35 years old OR (95% CI)	>35 years old OR (95% CI)		
Model 1^a^					

Metabolic syndrome	1.15 (1.04–1.26)	1.80 (1.12–2.91)	3.76 (1.53–9.22)	<0.001	0.007
Individual component of metabolic syndrome					
Elevated waist circumference	1.27 (1.17–1.38)	2.11 (1.39–3.19)	3.18 (1.31–7.73)	<0.001	0.007
Elevated blood pressure	1.47 (1.35–1.60)	2.31 (1.50–3.55)	2.03 (0.82–5.05)	<0.001	0.103
Elevated triglyceride	1.07 (0.97–1.18)	1.06 (0.63–1.77)	2.64 (1.06–6.55)	0.149	0.151
Elevated fasting glucose	1.10 (1.00–1.22)	1.38 (0.84–2.26)	0.69 (0.20–2.41)	0.154	0.527
Reduced HDL cholesterol	1.02 (0.94–1.11)	1.34 (0.88–2.04)	4.83 (2.00–11.7)	0.008	0.001

Model 2^b^					

Metabolic syndrome	1.19 (1.09–1.32)	1.96 (1.20–3.21)	4.26 (1.59–11.4)	<0.001	0.008
Individual component of metabolic syndrome					
Elevated waist circumference	1.32 (1.21–1.43)	2.18 (1.43–3.35)	3.54 (1.36–9.28)	<0.001	0.008
Elevated blood pressure	1.50 (1.38–1.64)	2.40 (1.55–3.72)	2.02 (0.79–5.18)	<0.001	0.110
Elevated triglyceride	1.08 (0.98–1.20)	1.17 (0.69–1.97)	2.50 (0.96–6.48)	0.142	0.164
Elevated fasting glucose	1.13 (1.02–1.25)	1.40 (0.84–2.33)	0.68 (0.19–2.52)	0.156	0.566
Reduced HDL cholesterol	1.03 (0.94–1.12)	1.46 (0.95–2.24)	5.58 (2.18–14.3)	0.008	0.002

Model 3^c^					

Metabolic syndrome	1.20 (1.09–1.32)	2.12 (1.29–3.49)	4.38 (1.62–11.9)	<0.001	0.007
Individual component of metabolic syndrome					
Elevated waist circumference	1.32 (1.22–1.44)	2.26 (1.47–3.46)	3.81 (1.44–10.0)	<0.001	0.006
Elevated blood pressure	1.51 (1.38–1.64)	2.41 (1.55–3.74)	1.97 (0.77–5.09)	<0.001	0.116
Elevated triglyceride	1.08 (0.98–1.20)	1.25 (0.74–2.11)	2.52 (0.96–6.61)	0.104	0.153
Elevated fasting glucose	1.13 (1.03–1.25)	1.46 (0.87–2.44)	0.64 (0.17–2.39)	0.098	0.554
Reduced HDL cholesterol	1.03 (0.95–1.13)	1.47 (0.95–2.27)	5.50 (2.12–14.3)	0.006	0.002

CI, confidence interval; HDL, high-density lipoprotein; OR, odds ratio.

^a^Adjusted *P* values by age at enrollment.

^b^Adjusted *P* values by age at enrollment, age at first pregnancy, number of childbirths, BMI status before pregnancy, history of spontaneous abortion, history of artificial abortion, breastfeeding experience, use of oral contraceptives, and menopausal status.

^c^Adjusted *P* values by age at enrollment, age at first pregnancy, number of childbirths, BMI status before pregnancy, history of spontaneous abortion, history of artificial abortion, breastfeeding experience, use of oral contraceptives, menopausal status, education level, smoking status, drinking status, and physical exercise.

## DISCUSSION

A history of preeclampsia appears to be independently associated with increased odds of metabolic syndrome and its individual components, including central obesity, elevated blood pressure, and elevated fasting glucose level, in women after their first pregnancy. Additionally, women who first became pregnant at older ages and had preeclampsia were found to be at significantly increased likelihood of developing metabolic syndrome later in life.

Preeclampsia itself may contribute to subsequent risk of pathophysiological abnormalities related to cardiovascular diseases, which occur due to perturbations in multiple metabolic pathways. Although the sample in the present study was restricted to healthy gravida women who were free of any pre-existing metabolic disorders, they were found to be significantly more prone to developing metabolic disorders, such as central obesity, elevated blood pressure, elevated fasting glucose level, and other such conditions in later life, simply by having a pregnancy complicated by preeclampsia. Preeclamptic women are assumed to be at greater risk of poor health as they age, since metabolic syndrome is a crucial risk factor for cardiovascular disease incidence and mortality^21^ and the prevalence of metabolic syndrome tends to increase linearly with age, due to multiple age-related physiologic mechanisms.^22^ Further longitudinal follow-up studies will help to determine the long-term effect of preeclampsia and elucidate hidden associations between preeclampsia, cardiovascular disease, and metabolic syndrome.

The present results are consistent with previous studies reporting that preeclamptic women tended to have higher BMI and blood pressure and unfavorable lipid profiles, which are known to be risk factors for cardiovascular disease in later life,^23^^,^^24^ and that the prevalence of metabolic syndrome was significantly higher in women who had experienced a pregnancy complicated by preeclampsia.^18^^,^^25^ Additionally, a previous meta-analysis reported that women diagnosed with hypertensive pregnancy disorders, such as preeclampsia, eclampsia, and gestational hypertension, were at a higher risk of having subsequent poor biochemical cardiovascular indicators (ie, levels of triglycerides, HDL cholesterol, glucose, insulin, and microalbumin) than women with normotensive pregnancies.^26^ This indicates that abnormal metabolic alterations activated and/or induced by a preeclamptic pregnancy may affect the development of cardiovascular diseases in the future. Further, given that pregnancy is a metabolic and vascular ‘stress test’ for women,^14^ a woman diagnosed with preeclampsia (ie, a woman who fails the stress test) may be assumed to be more prone to cardiovascular-related complications caused by metabolic disorders over her lifetime than women with normal pregnancies.^14^^,^^16^

Advanced maternal age appears to provoke a negative effect of preeclampsia on risk of subsequent development of metabolic syndrome. In the present study, we attempted to evaluate both the independent and synergistic effects of preeclampsia and late age at first pregnancy on subsequent metabolic disease risks over the lifetime of gravida women. In contrast with preeclampsia, maternal age itself was found to be inversely associated with risk of metabolic syndrome later in life (data not shown). This is consistent with previous findings from Korea that postmenopausal women who had their first child at an older age were at lower risk of developing metabolic syndrome.^19^ This finding is probably best explained by the fact that advanced maternal age is associated with decreased parity and/or number of pregnancies; therefore, women who first become pregnant at older ages are less likely to be exposed to the detrimental effects (ie, insulin resistance, weight gain, and dyslipidemia) that can be induced by accumulated pregnancies, thereby reducing the risk of metabolic syndrome in later life.^19^^,^^27^^,^^28^

Interestingly, our results show that the protective effects of late age at first pregnancy on metabolic health in middle-aged women dissipate if the women become pregnant at an older age and experience a pregnancy complicated by preeclampsia. Preeclamptic women who first became pregnant at age >35 years were found to have a significant increase in their adjusted risk of metabolic syndrome compared to women younger than 35 at first pregnancy without preeclampsia. Although the results were somewhat hindered by the small number of women exposed to both conditions (ie, preeclampsia and late age at first pregnancy), our findings indicate that preeclampsia and late age at first pregnancy could be linked and act as crucial risk factors in both a dependent and/or independent manner in the etiology of future metabolic syndrome. Experimental and longitudinal studies focused on biological changes and interactions in the female reproductive system during pregnancy will further clarify the causal mechanisms underlying the long-term effects of maternal age, preeclampsia, and future metabolic health in women.

A certain number of metabolic and vascular alterations generally occur due to the developing placenta and fetus; however, preeclamptic women who also have endothelial damage and dysfunction tend to be under excessive metabolic stress.^16^ Moreover, the features of preeclampsia, including insulin resistance, hypertension, and unfavorable lipid profiles, overlap with those of metabolic syndrome. This implies that the basis of preeclampsia depends on metabolic disturbances and that the excessive metabolic stress that occurs during gestation could be retained and/or turn into metabolic syndrome after pregnancy. Based on a literature review^11^^–^^14^^,^^16^^,^^21^^,^^24^ and the present results, it is possible to infer that: 1) metabolic stress, whether it is a new-onset condition induced by pregnancy or an intensified predisposition throughout pregnancy, appears to be an overt antecedent of metabolic syndrome in later life; 2) metabolic stress during pregnancy in women who first become pregnant at an age over 35 years is more likely to have a severe impact on future metabolic health; and 3) women who have both a history of preeclampsia and first pregnancy at an older age may be vulnerable and more likely to experience metabolic syndrome and cardiovascular disease in the future.

Several limitations to this study should be noted. First, preeclampsia ascertainment was based on self-reported information; therefore, this study may not be completely free of misclassification and/or recall bias. If a large proportion of the preeclampsia-positive women were missed, or if silent cases were misclassified into the preeclampsia-negative group, the results could be biased. However, given that these effects would tend toward the null hypothesis and attenuate the magnitude of risk estimates in the observed associations, the findings of the present study should be relatively conservative estimates of risks. Furthermore, to ensure the reliability of the self-reported information, percent agreement of 570 subjects who re-visited within 4 years after the first enrollment was analyzed. The percent agreements of preeclampsia history and parity were relatively high (94% and 96%, respectively), and those of other reproductive factors reached around 80% or more, signifying a fairly high level of reliability. Second, we could not explore the pathophysiology of preeclampsia due to a lack of detailed information on preeclampsia, including severity, time of onset, and other comorbid symptoms. Moreover, though the subsequent risk of metabolic syndrome might be affected by various pregnancy-related disorders as well as preeclampsia itself, other pregnancy complications (ie, eclampsia, gestational diabetes mellitus, and hypertension) could not be assessed in the present study. Therefore, our findings should be interpreted with caution. Finally, the cross-sectional design did not allow us to firmly establish a causal relationship. However, we followed the exposed (ie, preeclampsia-positive) and non-exposed (ie, preeclampsia-negative) subjects over time, and after confirming the development of new-onset metabolic diseases, we selected women who did not have pre-existing metabolic disorders before their first pregnancy and estimated all associations as multivariable odds ratios. Therefore, temporal ambiguity—the major flaw of cross-sectional study designs—is diminished and our results have logical plausibility.

In the present study, we demonstrated that preeclampsia in women who first become pregnant at an older age could increase the risk of developing metabolic syndrome later in life. To screen women who are at high risk of metabolic disorders and cardiovascular diseases later in life, clinicians should identify women who have experienced preeclamptic pregnancy or who first become pregnant at older ages. Women who fit both of these criteria should be treated as having an increased risk of future cardiovascular disease. Our findings suggest that, to promote the cardiovascular and metabolic health of women, these women at increased risk should be targeted for both tailor-made interventions for risk modification and closer surveillance.
